# Exploring Voice Acoustic Features Associated with Cognitive Status in Korean Speakers: A Preliminary Machine Learning Study

**DOI:** 10.3390/diagnostics14242837

**Published:** 2024-12-17

**Authors:** Jiho Lee, Nayeon Kim, Ji-Wan Ha, Kyunghun Kang, Eunhee Park, Janghyeok Yoon, Ki-Su Park

**Affiliations:** 1 Neopons Inc., Daegu 41260, Republic of Korea; jiholee255@neopons.com (J.L.); nancy@neopons.com (N.K.); 2 Department of Speech-Language Pathology, Daegu University, Gyeongsan 38453, Republic of Korea; jw-ha@daegu.ac.kr; 3 Department of Neurology, School of Medicine, Kyungpook National University, Daegu 41404, Republic of Korea; kangkh@knu.ac.kr; 4 Department of Rehabilitation Medicine, School of Medicine, Kyungpook National University, Daegu 41404, Republic of Korea; ehmdpark@naver.com; 5 Department of Industrial Engineering, Konkuk University, Seoul 05029, Republic of Korea; 6 Department of Neurosurgery, School of Medicine, Kyungpook National University, Daegu 41404, Republic of Korea

**Keywords:** cognitive impairment, pre-screening, K-MMSE, speech features, speech tasks, deep learning

## Abstract

**Objective**: To develop a non-invasive cognitive impairment detection system using speech data analysis, addressing the growing global dementia crisis and enabling accessible early screening through daily health monitoring. **Methods**: Speech data from 223 Korean patients were collected across eight tasks. Patients were classified based on Korean Mini-Mental State Examination scores. Four machine learning models were tested for three binary classification tasks. Voice acoustic features were extracted and analyzed. **Results**: The Deep Neural Network model performed best in two classification tasks, with Precision-Recall Area Under the Curve scores of 0.737 for severe vs. no impairment and 0.726 for mild vs. no impairment, while Random Forest achieved 0.715 for severe + mild vs. no impairment. Several acoustic features emerged as potentially important indicators, with DDA shimmer from the /i/ task and stdevF0 from the /puh-tuh-kuh/ task showing consistent patterns across classification tasks. **Conclusions**: This preliminary study suggests that certain acoustic features may be associated with cognitive status, though demographic factors significantly influence these relationships. Further research with demographically matched populations is needed to validate these findings.

## 1. Introduction

The early detection and management of cognitive impairment have become critical challenges in modern healthcare systems [1,2,3,4]. Traditional diagnostic methods for cognitive disorders, particularly dementia, often rely on time-consuming clinical assessments and expensive neuroimaging techniques [5,6,7]. These approaches, while accurate, present significant barriers, including high costs, limited accessibility, and potential patient discomfort, especially for elderly individuals who may be reluctant to undergo extensive medical procedures. Consequently, there is an urgent need for more accessible and non-invasive screening tools that can support the early detection of cognitive decline.

Recent advances in artificial intelligence (AI) technology have opened new possibilities for the detection of cognitive impairment through various approaches. Many studies have demonstrated the effectiveness of AI applications using neuroimaging data such as MRI and PET [8,9,10], which provide detailed structural and functional brain information. While these methods offer high diagnostic accuracy, their widespread implementation may be limited by the need for specialized equipment and facilities. As a complementary approach, speech analysis has emerged as a promising direction, as speech production involves complex cognitive processes including attention, memory, language construction, and motor control [4,11,12]. These different approaches each offer unique advantages for cognitive assessment, potentially serving different clinical needs and contexts.

A robust body of research has investigated the relationship between vocal characteristics and cognitive function, suggesting that certain acoustic features could potentially indicate early signs of cognitive decline. Quatieri et al. (2020) [13] found that voice perturbation features, such as jitter (frequency variability) and shimmer (amplitude variability), can effectively identify early signs of cognitive impairment. Lin et al. (2020) [14] and Robin et al. (2020) [15] have further explored these features, showing significant associations between shimmer patterns and cognitive impairment severity, as well as the utility of these parameters in detecting cognitive changes before they become clinically apparent. Expanding on this, Thomas et al. (2021) [16] identified shimmer as a sensitive indicator of advanced cognitive impairment, while Zhao et al. (2022) [17] highlighted the progressive nature of vocal changes, suggesting that these patterns could reflect different stages of cognitive deterioration.

Beyond jitter and shimmer, other studies have analyzed various acoustic features that contribute to our understanding of cognitive health. For instance, M Simões-Zenari et al. (2022) [18] examined harmonic-to-noise ratio (HNR) as an indicator of vocal clarity, finding that reduced HNR values correlated with early cognitive impairment due to degraded neuromuscular control. Similarly, Pierce et al. (2019) [19] studied prosodic features such as pitch variability and speech rate, which have been linked to diminished cognitive function and are particularly useful in distinguishing between mild and severe impairment. Further, T Pommée et al. (2021) [20] observed changes in formant frequencies in patients with cognitive decline, particularly in higher formants that reflect articulatory precision, which tends to diminish as cognitive function declines.

Our research contributes to the field in three ways. First, while previous studies have primarily focused on English speakers, we specifically analyze speech patterns in Korean patients, accounting for language-specific characteristics in cognitive assessment. Second, we employ a comprehensive set of speech tasks combined with extensive acoustic feature extraction (184 indicators from 8 tasks), enabling a more thorough analysis of the speech patterns that may be associated with cognitive impairment. Third, through the application of SHAP analysis on machine learning models, we explore the relative importance of various acoustic features (such as shimmer and jitter parameters) in relation to different levels of cognitive status, providing insights for potential screening applications.

The objectives of our study are threefold:

1. To develop and evaluate a machine learning-based classification system for detecting cognitive impairment using speech data from multiple vocal tasks.
2. To investigate specific acoustic features that may be associated with different levels of cognitive impairment in Korean speakers.
3. To explore the feasibility of developing practical, non-invasive screening tools that could potentially be implemented in various healthcare settings.

This preliminary research suggests potential contributions to both technical and clinical aspects of cognitive assessment. From a technical perspective, our exploratory approach indicates the feasibility of combining multiple vocal tasks with advanced AI analysis techniques. Clinically, these initial findings may provide insights toward developing accessible screening tools that could enable regular monitoring of cognitive function in daily life settings, potentially supporting the early detection and management of cognitive decline.

## 2. Materials and Methods

### 2.1. General Overview

**Figure 1:**
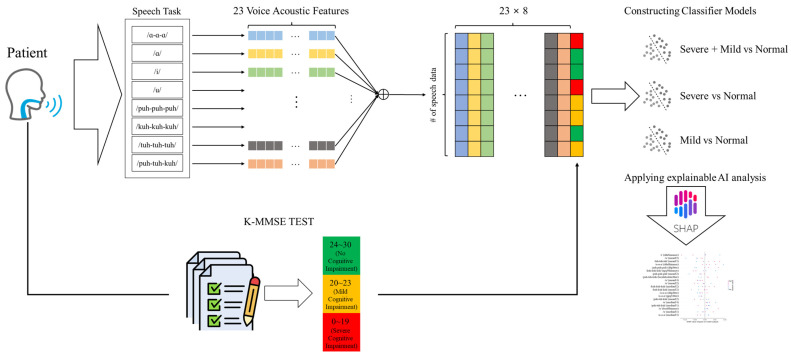
Overview of the proposed approach.

The research process in the present study consisted of the following steps (Figure 1):

- Collection of speech data from 223 patients with suspected cognitive impairment and extracting voice acoustic features.
- Grouping of patients based on K-MMSE scores to assess cognitive status.
- Development of machine learning models to classify patients into cognitive status groups.
- Examination of voice characteristics related to cognitive status through explainable AI analysis.

### 2.2. Data Preprocessing

#### 2.2.1. Patients

The study included participants who met the following criteria:

- Korean patients visiting the neurosurgery clinic with suspected cognitive impairment.
- Ability to understand and follow task instructions.
- No history of other neurological conditions affecting speech.
- Provided informed consent for participation in the study.

A total of 223 Korean patients with suspected cognitive impairment participated in the present study. All participants underwent initial screening by a speech-language pathologist with over 5 years of experience to ensure they could appropriately complete the speech tasks. The screening confirmed participants’ ability to:

- Understand and follow task instructions.
- Produce intelligible speech.
- Complete all required speech tasks.

All patients completed the K-MMSE under the observation of a medical professional. The K-MMSE is a neuropsychological test designed to assess cognitive function that has been adapted to the Korean language and culture [21]. Originally developed in the United States, the MMSE has been widely used throughout the world, and the K-MMSE is its Korean version. The assessment evaluates areas such as time and place orientation, registration, attention and calculation, recall, language, and spatial and visual perception. Following established guidelines and previous research [22], patients were classified into three groups based on their K-MMSE scores: severe cognitive impairment (Severe group: 0–19 points), mild cognitive impairment (Mild group: 20–23 points), and no cognitive impairment (Normal group: 24–30 points). These ranges have been validated in previous Korean population studies and are widely used in clinical settings for cognitive assessment. All procedures were performed under the supervision of a neurologist.

The patient data are summarized in Table 1. Previous research has found evidence suggesting that an individual’s age and education level can affect their cognitive decline [23,24]. This trend was also observed among the patients who took part in this research. In particular, the average age of the severe group was 72.44 years, compared with 71.11 and 64.80 years for the mild and normal groups, respectively, while the years of education for the severe, mild, and normal groups were 7.10, 8.24, and 10.93, respectively. However, we did not control for the age and educational background of the groups because we sought to pre-screen cognitive impairment. Although cognitive decline may be a result of aging, it can also be a precursor to dementia. Therefore, it is justifiable to identify cognitive decline in all cases during the pre-screening process.

#### 2.2.2. Speech Data Collection

Speech production involves complex cognitive processes requiring coordinated motor control and planning abilities. Our study utilized two complementary assessment approaches to comprehensively evaluate these processes: vowel tasks and diadochokinetic (DDK) tasks [25].

Our protocol consisted of eight distinct speech tasks:

1. Vowel Tasks (4 tasks)
  - Sustained vowel production (/α/, /i/, /u/): Participants sustained each vowel for 2–3 s to assess phonatory stability and fundamental vocal control.
  - Vowel prolongation (/α-α-α/): Participants sustained /α/ for as long as possible to evaluate maximum phonation time and respiratory control.
2. DDK Tasks (4 tasks)
  - Alternate motion rate (AMR) tasks (/puh-puh-puh/, /tuh-tuh-tuh/, /kuh-kuh-kuh/): Participants rapidly repeated individual syllables to assess speech-motor function and coordination [26].
  - Sequential motion rate (SMR) task (/puh-tuh-kuh/): Participants produced a sequence of different syllables to evaluate motor planning and sequencing abilities [27].

The inclusion of both vowel and DDK tasks was specifically designed to provide complementary information about different aspects of speech motor control [28]. Vowel tasks focus on assessing the stability and control of basic phonatory function, providing information about fine motor control of the vocal mechanism. These tasks generate clear tone samples that are particularly suitable for the acoustic analysis of voice stability parameters.

In contrast, DDK tasks evaluate higher-level motor planning and execution through rapid, repetitive movements [29]. These tasks are particularly sensitive to cognitive-motor impairment as they require the intact functions of motor planning, sequencing, and timing control. The AMR tasks assess the ability to repeatedly produce a single articulatory movement, while the SMR task evaluates the ability to plan and execute sequences of different articulatory movements.

This dual-task approach enables comprehensive assessment of both basic vocal function and complex motor speech abilities, potentially providing more sensitive indicators of cognitive-motor impairment than either approach alone [28]. All tasks were performed in a controlled environment following standardized protocols to ensure consistent and reliable data collection.

Speech data collection was conducted following standardized protocols in a controlled environment designed specifically for high-quality voice recording. The ambient noise was maintained below 50 dB to ensure optimal recording conditions [30,31]. A speech-language pathologist with over 5 years of experience conducted all voice sampling sessions.

**Figure 2:**
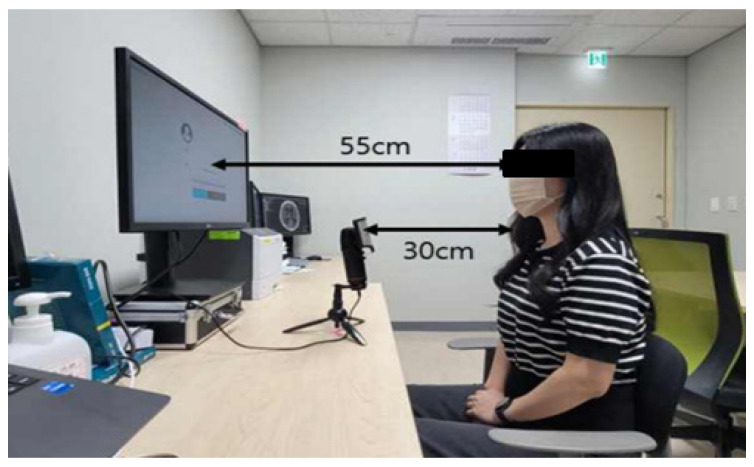
Environmental setup for speech data collection.

Based on established voice recording methodologies, we maintained consistent recording conditions: patients stood 55 cm from a monitor displaying task instructions, with a unidirectional microphone positioned 30 cm from their mouth (Figure 2). This standardized positioning ensures optimal voice capture while minimizing environmental variations. The speech data were digitally recorded at a sampling frequency of 44,100 Hz and saved in wav format. This sampling rate was chosen based on the Nyquist theorem, ensuring the accurate capture of the full range of human voice frequencies without aliasing [32]. Since the human voice typically contains frequencies up to about 20 kHz, our sampling rate of 44,100 Hz adequately captures all relevant acoustic features for analysis.

#### 2.2.3. Extracting Voice Acoustic Features

In human speech, a voice waveform can exhibit variable patterns, thus it is essential to evaluate irregularities in pitch and amplitude. Quantifying perturbations and noise levels in the voice is also important. Various voice acoustic features have been employed to objectively measure variation and noise levels in the voice. The human voice is a composite wave formed by the amalgamation of simple waves and exhibits periodicity. The lowest frequency of a periodic wave is referred to as the fundamental frequency (F0). Voice acoustic features can be categorized as voice quality or prosody features. The former group assesses the periodicity and uniformity of speech, while the latter group focuses on formant frequency patterns.

This study investigated 23 voice acoustic features based on an extensive review of the literature (Table 2).

The voice quality features were jitter, shimmer, and the harmonic-to-noise ratio (HNR). Jitter quantifies the variability in F0 between consecutive cycles within a specific timeframe. It is synonymous with the frequency variation rate or frequency perturbation (Equation (1)).

(1) $$Jitter=\left(\frac{\sum \left|P_{i}-P_{i-1}\right|}{n-1}\right)÷\bar{P}\times 100,\;$$

where P_i_ is the duration of the ith period, P_i−1_ is the duration of the (_i−1_)th period, *n* is the number of periods analyzed, and $\bar{P}$ is the average period duration.

Because jitter indicates the variation between successive cycles, jitter values are higher for more unstable voices [33]. Jitter calculates the rate of change between adjacent pitch periods. Depending on the number of adjacent intervals considered, various modifications to the jitter metric are possible. For example, local absolute jitter reflects the absolute change in the pitch between adjacent periods [34], relative average perturbation (RAP) jitter represents the average change in the pitch period over two neighboring intervals [35], period perturbation quotient (PPQ5) jitter is the average rate of the change in pitch over four contiguous analysis intervals [36], and difference of differences of periods (DDP) jitter is the mean change in the pitch period across consecutive intervals [37].

Shimmer quantifies the variability in the amplitude between consecutive cycles over a specified duration [38]. It is also referred to as the amplitude variation rate or amplitude perturbation (Equation (2)).

(2) $$Shimmer=\left(\frac{\sum \left|A_{i}-A_{i-1}\right|}{n-1}\right)÷\bar{A}\times 100,\;\;\;\;\;$$

where A_i_ is the amplitude of the ith period, A_i−1_ is the amplitude of the (_i−1_)th period, *n* is the number of periods analyzed, and $\bar{A}$ is the average amplitude.

A higher shimmer value indicates higher instability in the voice [39]. Various shimmer measurements are possible, allowing amplitude disruptions over varying numbers of periods to be investigated or different methods to be employed. For example, difference of differences of amplitude (DDA) shimmer is similar in concept to the DDP. DDA shimmer calculates the average absolute difference between consecutive differences in amplitudes across a set number of cycles [40]. The amplitude perturbation quotient for 5 cycles (APQ5) measures the amplitude variability using a 5-cycle window [41]. Specifically, APQ5 shimmer is the average absolute difference between consecutive amplitudes over these five cycles, divided by the average amplitude of those cycles. Similarly, APQ3 and APQ11 represent the mean amplitude variability over three and eleven cycles, respectively [42]. Local decibel (dB) shimmer is the mean absolute difference between consecutive dB amplitude values [43]. Instead of directly working with the raw amplitude values, local dB shimmer converts them to dB and then evaluates the variability.

The HNR is an objective measure of voice clarity. A higher HNR indicates a voice with minimal noise interference, while a lower HNR indicates a voice with significant noise components (Equation (3)).

(3) $$HNR=10\times \log_{10}\frac{Energy\;of\;Harmonics}{Energy\;of\;Noise}$$

In Equation (3), the harmonics are integer multiples of F0. For example, if a voice has an F0 of 100 Hz, the second harmonic is 200 Hz (2F0), and the third harmonic is 300 Hz (3F0). The energy of these harmonics is a measure of the squared magnitude of a signal integrated over time (Equation (4)). Based on Equations (3) and (4), the HNR measures the proportion of periodic energy compared with random noise within a signal. The HNR thus provides valuable information about voice quality and clarity.

(4) $$Energy\;of\;Harmonics=\sum_{n=1}^{N}{\left|H_{n}\right|}^{2},\;\;\;\;\;\;\;$$

where N is the number of harmonics considered and $H_{n}$ is the amplitude of the nth harmonic.

The prosody features listed in Table 2 are related to voice frequency and duration. Duration refers to the length of speech sounds. In a controlled setting, such as during a designated speech task, the duration of speech may vary depending on the patient’s condition. In addition, speech frequency features are characteristics of a speech signal that can be analyzed and measured in the frequency domain. These features capture the spectral properties of a voice and are employed in many applications, such as speech processing, speech recognition, and clinical voice assessment [44,45,46]. Voice frequency features are generally derived from F0, including its mean and standard deviation (stdev).

In this study, 23 acoustic features were derived for each of the eight speech tasks using Python 3.10.14, leading to a total of 184 indicators for the assessment of the severity of cognitive impairment. These features were extracted from two distinct types of voice samples: clear tone samples and speech samples. Clear tone samples were obtained from sustained vowel tasks (/α/, /i/, and /u/) and the vowel prolongation task (/α-α-α/), where participants produce stable, continuous phonation to evaluate basic vocal fold vibration and voice stability. Speech samples came from DDK tasks (/puh-puh-puh/, /tuh-tuh-tuh/, /kuh-kuh-kuh/, and /puh-tuh-kuh/), which involve more complex articulation movements and assess motor speech control.

Traditional voice analysis typically examines these samples separately, focusing on fundamental frequency (F0) for clear tone samples and speaking characteristics for speech samples. However, our machine learning methodology enables analysis of how these different voice characteristics interact and collectively indicate cognitive decline. This comprehensive approach allows us to identify significant features that emerge from the complex relationships between various speech features, rather than analyzing each type of voice sample in isolation. By learning patterns from both individual feature contributions and their interactions, our models provide a more complete understanding of the voice changes associated with cognitive impairment.

### 2.3. Model Development Process

#### 2.3.1. Model Selection

We classified the patients into three groups (Severe, Mild, or Normal) and framed our research as three binary classification problems: Severe + Mild vs. Normal, Severe vs. Normal, and Mild vs. Normal). To classify the severity of cognitive impairment, we tested a series of different models, including a logistic linear classifier model (LM), a random forest (RF) model, a gradient boosting model (GBM), and a deep neural network (DNN), because they have been widely used for binary classification problems in past research [47,48,49]. Due to the range of options available, it has become important to test the algorithms used for disease diagnosis against each other, because insufficient data increases the risk of those models with many parameters overfitting the data, while identifying potential voice features can be difficult when applying a large pre-trained model to medical data.

In recent years, various deep learning architectures, such as transformers, convolutional neural networks, and residual networks, have attracted clinical interest for use in cognitive diagnostic applications due to their effective classification performance [50]. However, the traditional machine learning algorithms (LM, RF, GBM, and DNN) were employed in the present study because there were insufficient data to implement more advanced deep learning algorithms and because the voice acoustic features were in numerical form. In particular, in the severe vs. normal classification task, data from 72 and 97 patients, respectively, were used, while there were 126 and 97 patients in the severe + mild vs. normal comparison and 54 and 97 patients in the mild vs. normal comparison, respectively. In addition, spectrograms can be used to visually represent the change in frequency over time in voice data. However, because the goal of this study was to detect differences in the voices of patients based on their level of cognitive impairment, we analyzed acoustic features rather than spectrograms.

#### 2.3.2. Model Construction and Evaluation

To construct the classification models, this study utilized an automated machine learning (AutoML) system to determine the ideal model hyperparameters. AutoML reduces the time required to tune the hyperparameters by exploring various combinations to obtain the set that produces the most effective performance and has been proven to be effective by many previous studies [51,52,53,54].

**Figure 3:**
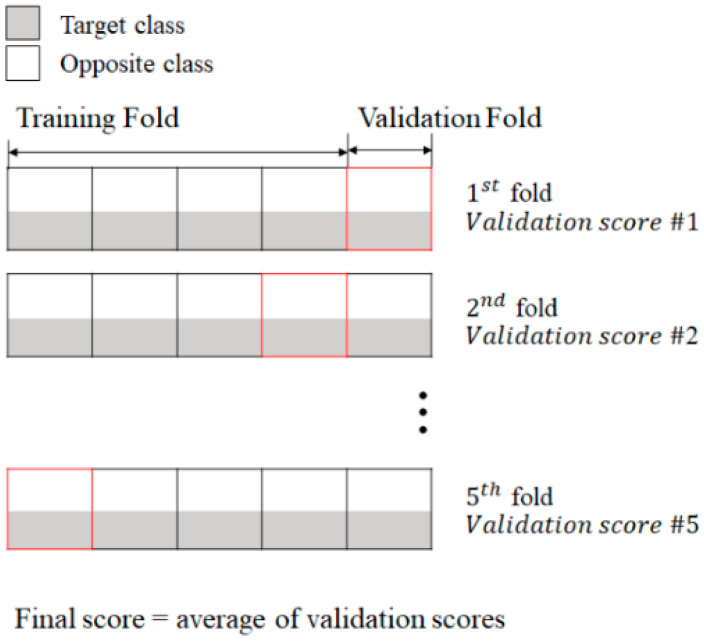
Summary of the five-fold cross-validation process.

A five-fold cross-validation approach was also utilized to address overfitting (Figure 3). In this process, the data set was divided into five groups, with one group sequentially serving as the validation set and the remaining four as the training set. This approach enables a more generalized evaluation of model performance across multiple data segments. For every classification task, the training and testing data were split at a ratio of 8:2.

The four classification models were tested for each of the three binary classification problems (severe vs. normal, severe + mild vs. normal, mild vs. normal). Due to the imbalanced data set for classification and limited testing data, it is not recommended to assess model performance using threshold-dependent metrics such as precision and recall. Therefore, to determine the highest-performing model, we calculated the precision–recall area under the curve (PR-AUC) score, which is widely used to evaluate models trained using imbalanced data [55]. The PR-AUC score was obtained by calculating the area under the curve (AUC) produced by plotting a model’s precision against its recall for various thresholds. The baseline PR-AUC score was calculated using Equation (5). If a model is successfully trained, its PR-AUC score should be higher than this baseline score.

(5) $$PR-AUC_{BASE}=\frac{Number\;of\;positive\;samples}{Number\;of\;all\;samples}$$

#### 2.3.3. Model Explanation

The pursuit of interpretable machine learning models has given rise to several techniques for interpreting their complex behavior. One of these is Shapley additive explanations (SHAP), which is grounded in cooperative game theory and provides a robust statistical framework for feature importance analysis [56]. For a game with a set N of players and value function $v:\;2^{N}\to \mathbb{R}$ that assigns a value to every coalition, the Shapley value $\phi_{i}\left(v\right)$ for player i is defined as shown in Equation (6) [57]:

(6) $$\phi_{i}\left(v\right)=\sum_{S\subseteq N\left\{i\right\}}\frac{\left|S\right|!\left(\left|N\right|-\left|S\right|-1\right)!}{\left|N\right|!}\left[v\left(S\cup \left\{i\right\}\right)-v\left(S\right)\right]$$

where S represents a coalition of players excluding player i. Equation (6) calculates the average contribution of feature i by considering all feasible combinations of features. This statistical approach calculates the average marginal contribution of each feature by considering all feasible combinations of features. When adapted to machine learning, the set N represents the features of the model, while value function v for subset S usually denotes the prediction of the model trained solely on S. The difference between the model’s output and average prediction can be parsed using the additive nature of SHAP values [56]. Therefore, SHAP values provide a statistically rigorous measure of feature importance by:

1. Quantifying the contribution of each feature to every possible model prediction.
2. Considering complex interactions between features.
3. Maintaining local accuracy and consistency.
4. Providing both global and local feature importance.

In our study, we utilized SHAP values to perform statistical analysis of voice features’ importance in cognitive impairment classification. This approach not only identifies potential voice features related to cognitive impairment but also quantifies their statistical contribution to the model’s predictions, providing a more comprehensive understanding than traditional univariate statistical tests. To identify potential features for cognitive impairment screening, we analyzed the 20 most important input voice features for each classification task (severe vs. normal, mild vs. normal, and severe + mild vs. normal) using SHAP values from the best performing model.

## 3. Results

### 3.1. Demographic Analysis

We first examined demographic characteristics across cognitive status groups to identify potential factors that should be considered when interpreting our findings. Table 3 and Table 4 present the demographic distributions between groups.

Non-parametric tests were employed due to non-normal distribution and heterogeneous variance. The analysis revealed significant group differences in both age (H = 24.07, *p* < 0.001) and education levels (H = 32.83, *p* < 0.001). Post hoc analysis showed that the normal group was significantly younger than both severe and mild groups, while no significant age difference was found between the severe and mild groups. Education levels showed a similar pattern, with the normal group having significantly higher education compared to both impairment groups. Gender distribution showed no significant differences between groups (χ^2^ = 2.79, *p* = 0.248).

### 3.2. Model Construction

This study constructed prediction models (LM, RF, GBM, and DNN) for the Severe vs. Normal, Severe + Mild vs. Normal, and Mild vs. Normal classification tasks. Table 5 summarizes the performance metrics for the models for each task. The performance metrics in Table 3 were calculated using the testing data.

In the Severe vs. Normal classification task, 72 patients were in the severe group and 97 were in the normal group. The DNN model produced the highest PR-AUC score at 0.737, which was almost twice as high as the baseline score (0.426). In the Severe + Mild vs. Normal classification task (151 and 72 patients, respectively), the RF was the best-performing model with a PR-AUC score of 0.715 (baseline = 0.677). Finally, in the Mild vs. Normal classification task (54 and 97 patients, respectively), the DNN outperformed the other models, nearly doubling the baseline PR-AUC score (0.726 vs. 0.358).

**Figure 4:**
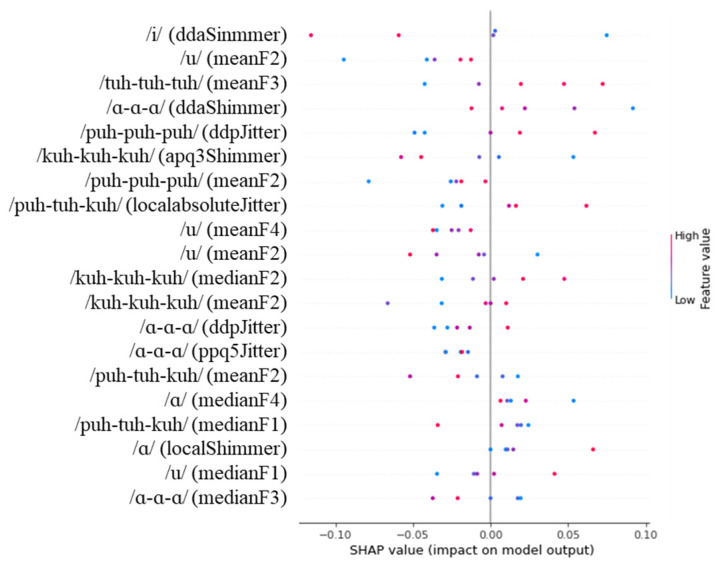
SHAP values for the severe vs. normal classification task.

**Figure 5:**
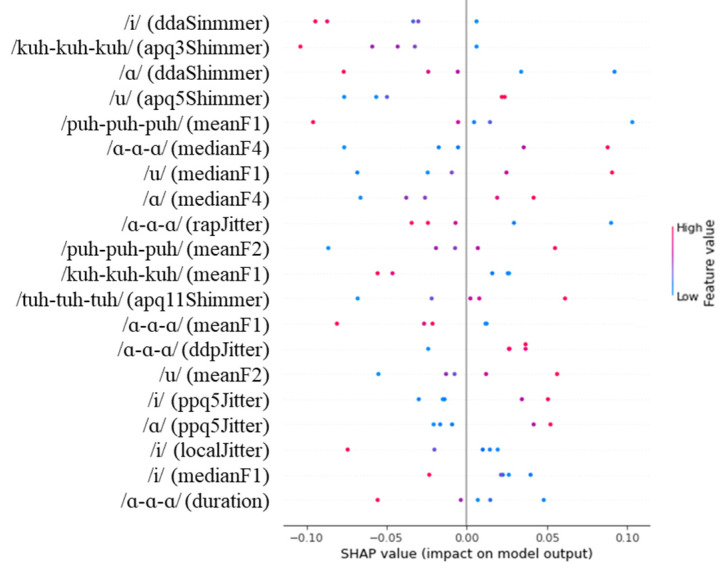
SHAP values for the mild vs. normal classification task.

**Figure 6:**
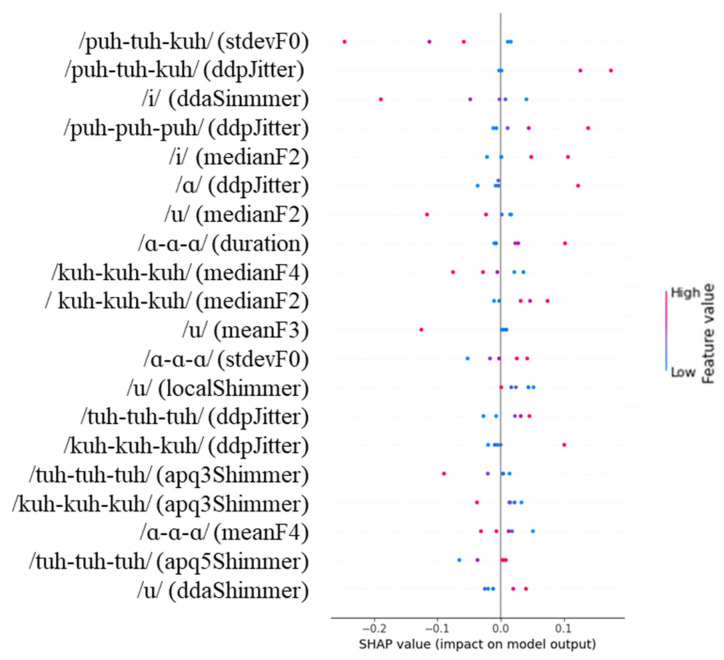
SHAP values for the severe + mild vs. normal classification task.

Figure 4, Figure 5 and Figure 6 show the SHAP values for the 20 most important features identified by our analysis. In the figures, red and blue indicate that the value of the feature is high and low, respectively. In addition, a positive SHAP value means that the model frequently uses it to predict the target, and a negative SHAP value means that the model frequently uses it to predict a non-target.

As shown in Figure 4, the feature that most strongly influenced the DNN model’s predictions for the Severe vs. Normal classification task was DDA shimmer, extracted from the /i/ task, with higher values for DDA shimmer associated with the prediction of normal group. In addition, the model predicted severe group when the mean F3 feature extracted from the /tuh-tuh-tuh/ task was higher, while the normal group was predicted when the value for DDA shimmer extracted from the /α-α-α/ task was higher. 

Similarly, as presented in Figure 5, DDA shimmer extracted from the /i/ task was the feature that had the most influence on the DNN model’s predictions for the mild vs. normal classification task, with higher values associated with normal group. The shimmer derivative features extracted from the /kuh-kuh-kuh/, /α/, and /u/ tasks were also found to have a significant influence. Specifically, the higher the apq5 shimmer feature extracted from the /u/ task, the more likely the model was to predict the mild group. Conversely, the shimmer derivative features extracted from the /kuh-kuh-kuh/ and /α/ tasks were positively associated with the prediction of mild group status.

As shown in Figure 6, for the severe + mild vs. normal classification task using the RF model, stdevF0 extracted from the /puh-tuh-kuh/ task was the feature that most strongly influenced the model’s predictions. Lower values of stdevF0 were associated with predicting cognitive impairment (severe + mild group), while higher values were linked to predictions of normal cognitive status. The ddaShimmer feature from the /i/ task also showed significant influence, where higher values were associated with predictions of cognitive impairment. Additionally, ddpJitter from the /puh-tuh-kuh/ task demonstrated notable importance, with higher values leading to predictions of cognitive impairment, while lower values were associated with normal cognitive status. The model also heavily weighted various formant-related features, including medianF2 from both /i/ and /u/ tasks, and medianF4 from the /kuh-kuh-kuh/ task. For these formant features, higher values generally corresponded to predictions of cognitive impairment. Furthermore, multiple shimmer derivatives, particularly apq3Shimmer from both the /tuh-tuh-tuh/ and /kuh-kuh-kuh/ tasks, showed consistent patterns where higher values were associated with predictions of normal cognitive status, while lower values suggested cognitive impairment.

## 4. Discussion

Our study’s primary finding is that certain acoustic features prioritized by our machine learning models align with previously reported vocal characteristics associated with cognitive decline. However, these results must be interpreted with significant caution due to several critical methodological considerations.

### 4.1. Interpretation of Machine Learning Results in the Context of Demographic Factors

The most crucial limitation of our findings stems from the substantial demographic differences between our study groups. As shown in Table 1, the normal group (mean age 64.80 years) was significantly younger than both the severe (72.44 years) and mild (71.11 years) groups. Similarly, education levels differed markedly, with the normal group averaging 10.93 years of education compared to 7.10 and 8.24 years in the severe and mild groups, respectively. These demographic disparities introduce important confounding factors that complicate the interpretation of our results.

The age difference is particularly concerning because aging naturally affects various acoustic parameters, independent of cognitive status. Prior studies have shown that older adults typically exhibit increased jitter and shimmer values, altered fundamental frequency patterns, and decreased voice stability due to physiological changes in the vocal apparatus [58,59]. Therefore, some of the acoustic features our models identified as important predictors may reflect age-related changes rather than cognitive decline specifically.

Educational background differences present another significant confounding factor. Education level has been shown to influence speech patterns through multiple mechanisms, including articulation precision, task comprehension, and overall cognitive reserve. The substantial disparity in educational attainment between our groups means that observed differences in acoustic features might partially reflect educational effects rather than purely cognitive status.

When examining our SHAP analysis results (Figure 4, Figure 5 and Figure 6), features like DDA shimmer from the /i/ task and APQ3 shimmer from the /kuh-kuh-kuh/ task emerged as influential predictors. However, without controlling for age and education either in the study design or through statistical methods, we cannot conclusively determine whether these features reflect cognitive decline or merely capture age-related changes in vocal production. The higher model performance in distinguishing severe from normal cases compared to mild from normal cases might partially result from larger demographic differences between these groups rather than purely cognitive factors.

These demographic confounds suggest that our findings should be interpreted primarily as proof-of-concept rather than definitive biomarkers of cognitive decline. Future research should address these limitations through more rigorous demographic controls, such as age-matched groups and education-stratified analyses. Longitudinal studies tracking changes within individuals over time would be particularly valuable in disentangling the effects of aging, education, and cognitive decline on vocal parameters. Additionally, statistical methods to adjust for demographic variables when analyzing acoustic features could help isolate cognitive status-specific effects from other influencing factors.

### 4.2. Methodological Considerations and Task-Specific Analysis

Our study employed a comprehensive protocol of eight distinct speech tasks, each designed to assess different aspects of vocal-motor control. The analysis of these tasks provided nuanced insights into how cognitive status may be reflected in various aspects of speech production.

#### 4.2.1. Sustained Vowel Tasks and Their Implications

The sustained vowel tasks (/α/, /i/, and /u/) were particularly informative in our analysis. Our SHAP analysis revealed that DDA shimmer from the /i/ task emerged as one of the strongest predictors across classification tasks. This finding is notable because the /i/ vowel requires precise control of tongue position and the maintenance of a relatively narrow vocal tract configuration [60,61]. The high predictive value of DDA shimmer in this task suggests that the fine motor control required for maintaining stable high front vowel production may be particularly sensitive to cognitive status.

However, the varying importance of shimmer features across different vowels raises important methodological questions. While DDA shimmer from /i/ showed high predictive value, similar features from /α/ and /u/ tasks showed different patterns of importance in our models. This task-specific variation suggests that certain articulatory configurations may be more sensitive to cognitive decline than others, though our current data cannot determine whether these differences reflect true cognitive-linguistic patterns or are artifacts of our sample’s demographic characteristics.

#### 4.2.2. DDK Tasks and Motor Control Assessment

The DDK tasks (/puh-puh-puh/, /tuh-tuh-tuh/, /kuh-kuh-kuh/, and /puh-tuh-kuh/) revealed distinct patterns in our analysis. Notably, APQ3 shimmer from the /kuh-kuh-kuh/ task emerged as a significant predictor in our models. This task involves repeated velar stop consonant production, requiring coordinated movements of the posterior portion of the tongue [25]. The importance of this feature might reflect the particular challenges of maintaining consistent amplitude during complex articulatory movements involving the posterior articulators.

The sequential motion rate task (/puh-tuh-kuh/) showed different acoustic patterns compared to the simple repetition tasks. Our analysis found that stdevF0 from this task was highly influential in the Severe + Mild vs. Normal classification (Figure 6). This finding may reflect the increased cognitive-motor demands of coordinating changes in place of articulation, as this task requires rapid transitions between labial, alveolar, and velar consonants [62].

#### 4.2.3. Model Performance Across Tasks

Our prediction models achieved different levels of performance across classification tasks:

- Severe vs. Normal: PR-AUC = 0.737.
- Mild vs. Normal: PR-AUC = 0.726.
- Severe + Mild vs. Normal: PR-AUC = 0.715.

These results demonstrate notable performance improvements over the calculated baseline scores (0.426, 0.358, and 0.677, respectively). Particularly, the severe vs. normal classification showed a 73% improvement over baseline, while the mild vs. normal classification achieved a remarkable 103% increase. The severe + mild vs. normal classification, despite having a higher baseline due to class distribution, still showed a 6% improvement. These substantial gains over baseline scores indicate our models’ robust ability to capture meaningful patterns in acoustic features for cognitive status prediction.

Previous studies have reported varying performance metrics using different approaches. Research using the Framingham Heart Study data achieved an AUC of 0.942 by combining acoustic features with linguistic and demographic variables [16]. Another study demonstrated that acoustic features alone could improve prediction performance, with AUC increasing from 0.773 (demographic features only) to 0.812 when acoustic features were included [14]. While these studies showed promising results, our study makes several unique contributions:

- Focused Acoustic Analysis: While previous studies often combined multiple feature types or showed modest improvements when adding acoustic features to demographic data, our study demonstrates the potential of using purely acoustic features for cognitive screening. This focused approach could be particularly valuable in situations where collecting linguistic or demographic data is challenging or impractical.
- Task-Feature Relationships: By analyzing specific acoustic features across different speech tasks, we identified which combinations of tasks and features are most indicative of cognitive decline. For example, our findings suggest that DDA shimmer from the /i/ task and stdevF0 from the /puh-tuh-kuh/ task are particularly informative, providing insights that could help optimize future screening protocols.
- Korean Language Context: Most previous studies have focused on English speakers, while our research provides specific insights into how cognitive decline manifests in Korean speech patterns. This contribution is particularly valuable given the potential differences in how cognitive impairment affects speakers of different languages.
- Severity-Specific Analysis: Rather than treating cognitive impairment as a binary condition, our approach differentiated between severe and mild impairment, providing more nuanced insights into how acoustic features might reflect different stages of cognitive decline.

While these performance results are encouraging, several important caveats must be considered:

1. The performance differences between classification tasks were relatively small.
2. The demographic differences between groups may have influenced these results.
3. The model’s performance should be compared with existing screening methods to establish practical utility.

These methodological variations underscore the importance of considering our results within their specific experimental context while acknowledging both the strengths and limitations of our focused acoustic feature approach.

#### 4.2.4. Feature Interactions and Task Dependencies

Our analysis revealed complex interactions between acoustic features and tasks. For instance, while shimmer-related features were important across multiple tasks, their relative importance varied depending on the specific task and classification problem. The prediction model identified different patterns of feature importance for severe vs. normal classification compared to mild vs. normal classification, suggesting that:

1. Different aspects of speech production may be affected at different stages of cognitive decline.
2. Certain task-feature combinations may be more sensitive to specific levels of cognitive impairment.
3. A multi-task approach might provide more robust assessment capabilities than single-task protocols.

However, these interpretations must be considered preliminary due to the limitations in our study design, particularly the lack of control for demographic factors and the absence of statistical validation of group differences in specific features.

#### 4.2.5. Clinical Feasibility Considerations

While our task battery showed promising results in terms of model performance, several practical considerations need to be addressed before clinical implementation:

1. The time required to complete all eight tasks may be burdensome for patients.
2. The technical requirements for accurate recording and analysis need to be standardized.
3. The relative contribution of each task to overall classification accuracy needs to be evaluated to potentially streamline the protocol.
4. The reliability and reproducibility of measurements across different clinical settings need to be established [63].

This detailed analysis of our task-specific findings suggests potential utility in using multiple speech tasks for cognitive assessment, while also highlighting the need for further validation and refinement of our approach. Future research should focus on determining which combinations of tasks and features provide the most reliable and efficient assessment while controlling for demographic and other confounding factors.

### 4.3. Limitations and Future Research Directions

While this preliminary study suggests promising directions for voice-based cognitive screening research, several important limitations should be considered. One notable consideration is the demographic differences between our study groups. The normal group (mean age 64.80 years) was younger than both the severe (72.44 years) and mild (71.11 years) groups, with similar patterns in education levels (normal: 10.93, mild: 8.24, severe: 7.10 years). These demographic variations suggest that future studies might benefit from controlling for age and education to better understand how these factors interact with voice characteristics in cognitive decline [58,59].

Our sample size (*N* = 223) provided sufficient data to demonstrate the potential of our approach while suggesting opportunities for more detailed analyses in future research. For instance, larger studies could explore gender-specific patterns in vocal characteristics and their relationship to cognitive status. The preliminary nature of our study focused on establishing feasibility rather than the statistical validation of specific biomarkers, laying groundwork for future investigations in this area.

Despite these limitations, our study contributes valuable insights to the field. The successful application of machine learning to analyze multiple speech tasks suggests a promising avenue for cognitive screening research. Our findings indicate that certain acoustic features, particularly from sustained vowel and DDK tasks, may merit further investigation as potential indicators of cognitive status. The relatively strong performance of our models, especially in distinguishing severe impairment (PR-AUC = 0.737) and mild impairment (PR-AUC = 0.726) from normal cognition, suggests that this approach could be worthy of further development.

Looking ahead, future research might explore several promising directions. Studies with demographically matched groups could help clarify the relationship between cognitive status and vocal features. Longitudinal investigations might provide insights into how these features change over time [64,65]. Additionally, cross-cultural and cross-linguistic studies could help determine whether certain vocal markers of cognitive status are universal or language-specific.

As voice-based screening tools continue to develop, our preliminary findings suggest that combining specific vocal tasks with machine learning analysis could contribute to the broader toolkit for cognitive assessment. While more research is needed to establish clinical utility, our study demonstrates the potential value of this approach and suggests promising directions for future investigation.

## 5. Conclusions

Our findings contribute to the growing body of literature on voice-based assessment of cognitive impairment, demonstrating the potential of acoustic features such as shimmer and jitter as indicators that warrant further investigation for non-invasive screening. By addressing the identified limitations and pursuing recommended future research directions, this study paves the way for integrating vocal biomarker analysis into routine cognitive health monitoring, particularly in accessible, everyday settings.

## Figures and Tables

**Table 1:** Cognitive impairment classification of the patients according to the K-MMSE.

Group	Age			Years of Education			Sex		
	Mean	SD	Range	Mean	SD	Range	Male	Female	Total
0 ≤ MMSE ≤ 19	72.44	8.57	46–87	7.10	4.49	0–16	32	40	72
19 < MMSE ≤ 23	71.11	8.36	48–84	8.24	4.05	0–16	31	23	54
23 < MMSE ≤ 30	64.80	11.30	30–85	10.93	4.13	0–16	43	54	97

**Table 2:** Voice acoustic features extracted from the collected speech data.

Category		Features	Description
Voice Quality		Jitter (PPQ5)	Period Perturbation Quotient: Average rate of pitch change over 4 contiguous analysis intervals
		Jitter (local)	Cycle-to-cycle variation in fundamental frequency; measures frequency instability in voice
		Jitter (DDP)	Difference of Differences of Periods: Mean change in pitch period across consecutive intervals
		Jitter (RAP)	Relative Average Perturbation: Average change in pitch period over two neighboring intervals
		Jitter (absolute)	Absolute change in pitch between adjacent periods
		Shimmer (DDA)	Average absolute difference between consecutive amplitude differences
		Shimmer (local)	Cycle-to-cycle variation in amplitude; measures amplitude instability in voice
		Shimmer (APQ5)	Amplitude Perturbation Quotient for 5 cycles: Average amplitude variability over 5 cycles
		Shimmer (localdb)	Mean absolute difference between consecutive dB amplitude values
		Shimmer (APQ3)	Amplitude Perturbation Quotient for 3 cycles: Mean amplitude variability over 3 cycles
		Shimmer (APQ11)	Amplitude Perturbation Quotient for 11 cycles: Mean amplitude variability over 11 cycles
		NHR	Harmonic-to-Noise Ratio: Measure of voice clarity; ratio of periodic to random energy
Prosody	Speech Rate	Duration	Length of speech sounds in controlled task settings
	Pitch	Avg. Formant	Average of the formant frequencies
		Mean F0	Average fundamental frequency of voice
		StDev F0	Variation in fundamental frequency
		Mean F1–F4	Average of first through fourth formant frequencies
		Median F1–F4	Median values of first through fourth formant frequencies

**Table 3:** Group comparisons of demographic characteristics.

Characteristic	Severe (*n* = 72)	Mild (*n* = 54)	Normal (*n* = 97)	Test Statistic	*p*-Value
Age, years	72.44 ± 8.57	71.11 ± 8.36	64.80 ± 11.30	H = 24.07 ^†^	<0.001
Education, years	7.10 ± 4.49	8.24 ± 4.05	10.93 ± 4.13	H = 32.83 ^†^	<0.001
Sex, *n* (%)				Χ^2^ = 2.79 ^‡^	0.248
- Male	32 (44.4%)	31 (57.4%)	43 (44.3%)		
- Female	40 (55.6%)	23 (42.6%)	54 (55.7%)		

Note: Values are presented as mean ± standard deviation unless otherwise specified. ^†^ Kruskal–Wallis H-test was used due to non-normal distribution (Shapiro–Wilk test, *p* < 0.05) and heterogeneous variance (Levene’s test for age: *p* = 0.017). ^‡^ Chi-square test.

**Table 4:** Post hoc analysis of between-group differences.

Comparison	*p*-Value *
Age	
- Severe vs. Mild	0.956
- Severe vs. Normal	<0.001
- Mild vs. Normal	0.003
Education	
- Severe vs. Mild	0.532
- Severe vs. Normal	<0.001
- Mild vs. Normal	<0.001

Note: * *p*-values for post hoc comparisons were adjusted using Bonferroni correction.

**Table 5:** Performance of the constructed classification models.

	Model	PR-AUC	AUC	Accuracy	F1	Precision	Recall
Severe vs. Normal group	DNN	0.737	0.716	0.618	0.666	0.542	0.867
	GBM	0.716	0.730	0.706	0.688	0.647	0.733
	LM	0.632	0.698	0.647	0.700	0.560	0.933
	RF	0.516	0.637	0.529	0.652	0.484	1.000
Normal vs. Mild + Severe group	RF	0.715	0.659	0.533	0.685	1.000	0.521
	GBM	0.682	0.651	0.573	0.686	0.921	0.547
	LM	0.680	0.637	0.560	0.692	0.974	0.536
	DNN	0.659	0.633	0.560	0.692	0.974	0.536
Mild vs. Normal group	DNN	0.726	0.794	0.667	0.688	0.524	1.000
	RF	0.630	0.785	0.800	0.727	0.727	0.727
	LM	0.597	0.636	0.500	0.571	0.417	0.909
	GBM	0.583	0.785	0.800	0.750	0.692	0.818

## Data Availability

The datasets presented in this article are not readily available because the data are part of an ongoing study.
